# Dual-function MIL-53(Al) incorporated cement composites for strength reinforcement and antibacterial activity

**DOI:** 10.1038/s41598-026-64478-9

**Published:** 2026-08-06

**Authors:** M. Ramadan, Ahmed Radwan, A. O. Habib, Alaa Mohsen

**Affiliations:** 1 Chemistry Department, Faculty of Science, Ain Shams University, Cairo, Egypt; 2 Faculty of Engineering, Ain Shams University, Cairo, Egypt

**Keywords:** Portland cement, Metal-organic frameworks, MIL-53(Al), Fresh and hardened properties, Antibacterial activity, Hospital buildings, Engineering, Materials science, Microbiology

## Abstract

This work presents the development of an antimicrobial cement with enhanced mechanical performance through the incorporation of metal-organic frameworks (MOFs) into the cement matrix. MIL-53(Al) was synthesized in the laboratory and thoroughly characterized using XRD, SEM, HR-TEM, and BET/BJH analyses. Cement pastes were then prepared by introducing MIL-53(Al) at dosages of 0.1, 0.3, 0.5, and 1 wt%. The resulting composites were evaluated in terms of workability, setting time, compressive strength, and antibacterial activity. The findings revealed that the addition of MIL-53(Al) reduces workability while accelerating both initial and final setting times. Mechanical testing demonstrated that a dosage of 0.5 wt% yields the most favorable results, increasing compressive strength by 43.8% after 1 day and 50.0% after 28 days of curing compared to the neat paste. Furthermore, the modified cement exhibited strong antibacterial performance against various strains, including *Bacillus subtilis*, *Staphylococcus aureus*, *Klebsiella pneumoniae*, and *Salmonella typhi*. Microstructural analyses using XRD and SEM/EDX confirmed that MIL-53(Al) plays a critical role as a filler and nucleation agent, promoting the formation of beneficial hydration products such as tobermorite, hydrogarnet, and strätlingite. These phases enhance matrix densification and load transfer, leading to improved mechanical performance.

## Introduction

The construction sector is increasingly embracing advanced materials to achieve greater sustainability, durability, and performance. Among these innovations, nanoparticles have proven to be highly effective in enhancing the mechanical, thermal, and functional characteristics of cement-based materials, coatings, and composites. Commonly studied nanoparticles include nano-silica^1^, nano-titanium dioxide^2,3^, and nano-alumina^4,5^, while carbon nanotubes (CNTs) are valued for their exceptional strength and conductivity, making them ideal for structural reinforcement and smart sensing applications^6^. Other nanomaterials, such as nano-clay^7^ and nano-iron oxide, contribute to improved thermal stability, accelerated hydration, radiation shielding, and antimicrobial resistance^8,9^. Collectively, these nanomaterials support the development of more durable and low-maintenance construction solutions.

In parallel, Metal-Organic Frameworks (MOFs) have gained attention as multifunctional additives in construction materials^10^. Recent studies show that engineering materials can perform better when their structure, interfaces, and stress behavior are carefully controlled^11–14^. Therefore, adding porous MOF to cement can be considered a similar strategy to improve strength, durability, and antimicrobial resistance. MOFs are crystalline porous compounds formed by the coordination of metal ions or clusters, known as secondary building units (SBUs), with organic ligands, resulting in highly ordered one-, two-, or three-dimensional networks^15^. Their exceptionally high surface area, tunable pore sizes, and chemical versatility make them ideal for applications in sensors^16,17^, gas storage, catalysis^18^, environmental remediation^19–22^, and increasingly, cement-based materials^23^. When incorporated into cementitious systems, MOFs influence both fresh and hardened properties. The incorporation of 0.6 wt% ZIF-67 nanoparticles into cement paste was found to reduce workability but significantly enhance compressive strength by 27.81% at 3 days and 20.36% at 28 days^10^. Zhang et al. ^24^ reported that adding 0.6 wt% ZIF-8 to Portland cement significantly improved compressive strength by 31% at 3 days and 16.2% at 28 days. ZIF-8 acts as a stable, non-reactive filler in alkaline environments, enhancing hydration and filling micro-voids, which contributes to the overall strength development of the cement. Yuan et al. ^23^ reported that adding 0.15 wt% of Zr(IV)-based MOF-808 to cement paste significantly enhanced compressive strength at early and later curing stages by 15.56% at 3 days and 13.03% at 28 days. MOF-808 also delayed the hydration process and reduced the heat released during hydration. MOF-808 is capable of chelating cement-derived ions such as Ca²⁺, Fe³⁺, and Al³. Its capacity to adsorb calcium ions is notably enhanced as its crystallinity decreases. The addition of 0.5 wt% ZIF-67 to cement paste resulted in boosting tensile strength by 113% and compressive strength by 44.8% after 28 days of curing^25^. MIL-101(Fe) and its amino-functionalized variant, NH₂-MIL-101(Fe), were found to accelerate cement hydration, refine cementitious porous system, and contribute to improved compressive strength^26^. The stability of MOF-5, HKUST-1, ZIF-8, UiO-66, and MIL-68(Al) was assessed in simulated cement pore environments. While MOF-5 and HKUST-1 showed structural degradation, ZIF-8, UiO-66, and MIL-68(Al) remained intact. Among them, ZIF-8 exhibited outstanding resistance to alkaline conditions, functioning as a stable filler and nucleation sites for extra growth of CSHs^27^. Cement composites incorporating specific MOFs like NH₂-MIL-125(Ti), UiO-66-(OH)_2_, COFs networks, and modified ZIF-8 have shown potential for various sustainability-focused applications, including CO_2_-capture^28^, heavy metal stabilization^29^, shrinkage reduction^30^, and enhanced corrosion resistance^31^.

MOFs interact with cement grains through a combination of physical and chemical mechanisms that contribute to the development of mechanical strength and refinement of microstructural morphology. Due to their high surface area and porous architecture, MOFs serve as effective nucleation sites for hydration products, particularly CSHs and CASHs, which accelerate the hydration process and promote a denser and more homogeneous microstructure^32^. Their metal centers (e.g., Zn²⁺, Al^3+^, Fe³⁺, Cr³⁺) can participate in ion exchange or catalytic reactions^33^, influencing the kinetics of cement hydration and facilitating the formation of stable hydration phases. Additionally, MOFs can adsorb free water and ions, modulate the rheology and reduce the formation of large capillary pores, which enhances packing density and reduces permeability^34^. The organic linkers within MOFs may also interact/chelate with cement components^35^, contributing to the stabilization of hydration products and limiting the growth of calcium hydroxide. These interactions collectively result in improved compressive and flexural strength, reduced porosity, and enhanced durability^36^. Furthermore, the uniform dispersion of MOFs within the cement matrix enhances antimicrobial activity^37^ and minimizes microcracking and shrinkage, contributing to long-term structural integrity and resilience under environmental stressors^30^.

MOFs have emerged as promising materials in the field of antimicrobial research due to their unique structural properties and the ability to incorporate bioactive metal ions. The antimicrobial activity of MOFs is primarily attributed to the release of metal ions such as silver (Ag⁺), copper (Cu²⁺), and zinc (Zn²⁺), which can disrupt microbial cell membranes, interfere with enzyme function, and generate reactive oxygen species (ROS)^38,39^. For example, Ag-based MOFs like Ag-MOF-74 have demonstrated strong anti-bacterial effects against Escherichia coli and Staphylococcus aureus^40^. Similarly, ZIF-8, a zinc-based MOF, exhibits broad-spectrum antimicrobial activity due to its ability to release Zn^2+^ ions and induce oxidative stress in microbial cells^41^. Chelation plays a pivotal role in the antimicrobial activity of MOFs, primarily through interactions between their metal ions and functional groups on microbial membranes, such as thiol, carboxyl, and phosphate moieties present in proteins and lipids. These interactions compromise membrane integrity, disrupt ion transport, and impair vital cellular functions^42^. Additionally, chelation facilitates the production of reactive oxygen species (ROS), which further damages microbial biomolecules, including DNA and proteins^43^. Beyond membrane interactions, the organic ligands within MOFs can chelate essential metal ions such as Mg²⁺, Ca²⁺, Zn²⁺, and Fe²⁺/Fe³⁺, which are critical for microbial enzymatic activity, metabolic pathways, and structural stability. By sequestering these ions, MOFs effectively inhibit processes like DNA replication, protein synthesis, and membrane maintenance, thereby enhancing their antimicrobial efficacy^42,44^.

Microbial colonization within cementitious systems poses a significant threat to their long-term durability and mechanical integrity^45^. The metabolic activities of bacteria and fungi can lead to the production of organic acids, sulfates, and other corrosive byproducts that degrade the cement matrix^46^. These microbial-induced reactions can accelerate pore formation, increase porosity, and weaken the interfacial bonding between hydration products, ultimately reducing compressive and tensile strength^47^. Additionally, biofilm formation on the surface of cement structures can trap moisture and promote localized deterioration, further compromising structural performance. Over time, these biological interactions contribute to premature aging, increased maintenance requirements, and reduced service life of concrete infrastructure, especially in humid or contaminated environments^48,49^.

Although MOFs have been extensively studied across various scientific domains, their integration as functional additives in cementitious systems to antimicrobial performance has not yet been thoroughly investigated. This study proposes a novel strategy by incorporating MIL-53(Al), synthesized via a modified hydrothermal method, into ordinary Portland cement (OPC) pastes. The unique physicochemical characteristics of MIL-53(Al), including its high surface area, facilitating an adaptable framework, make it an ideal candidate for improving fresh-state properties and promoting both mechanical strength and microbial resistance. By introducing MIL-53(Al) at varying concentrations (0.1, 0.3, 0.5, and 1 wt%), this study evaluates its influence on setting time, flowability, and compressive strength over a 28-day period. Furthermore, the antimicrobial efficacy of the modified cement was assessed using inhibition zone measurements around cement discs, following ASTM D4300-01 standards. To the best of our knowledge, this work represents a significant application of MIL-53(Al) in cement matrices, offering a new route toward multifunctional construction materials with enhanced hydration kinetics and improved resistance to microbial colonization.

## Experimental work

### Materials

Ordinary-Portland cement (OPC/CEM I/42.5 N) used in this work was sourced from the National Company for Cement, Beni Seuf, Egypt. Its oxide-composition was measured using X-ray fluorescence (XRF/Xios/model-PW-1400), as in Table 1. The Blaine surface area was determined to be 3800 cm^2^/g as measured by the Air Permeability apparatus (APT/HUMBOLDT/model-H3056.3 F). Aluminum chloride hydrate (AlCl_3_.6H_3_O), 1,4-benzenedicarboxylic acid (H_2_BDC), N,N-dimethylformamide (DMF) and glacial acetic acid (CH_3_COOH) were delivered from El-Nasr Company, Egypt, as commercial chemicals to prepare MIL-53(Al).

**Table 1:** Chemical oxide compositions for OPC (mass, %).

Material	SiO_2_	Al_2_O_3_	Fe_2_O_3_	CaO	MgO	SO_3_	Na_2_O	K_2_O	Cl^−^	LOI
OPC	20.69	4.67	3.51	64.11	1.27	2.92	0.29	0.22	0.02	2.30

### Synthesis and characterization of MIL-53(Al)

**Fig. 1:**
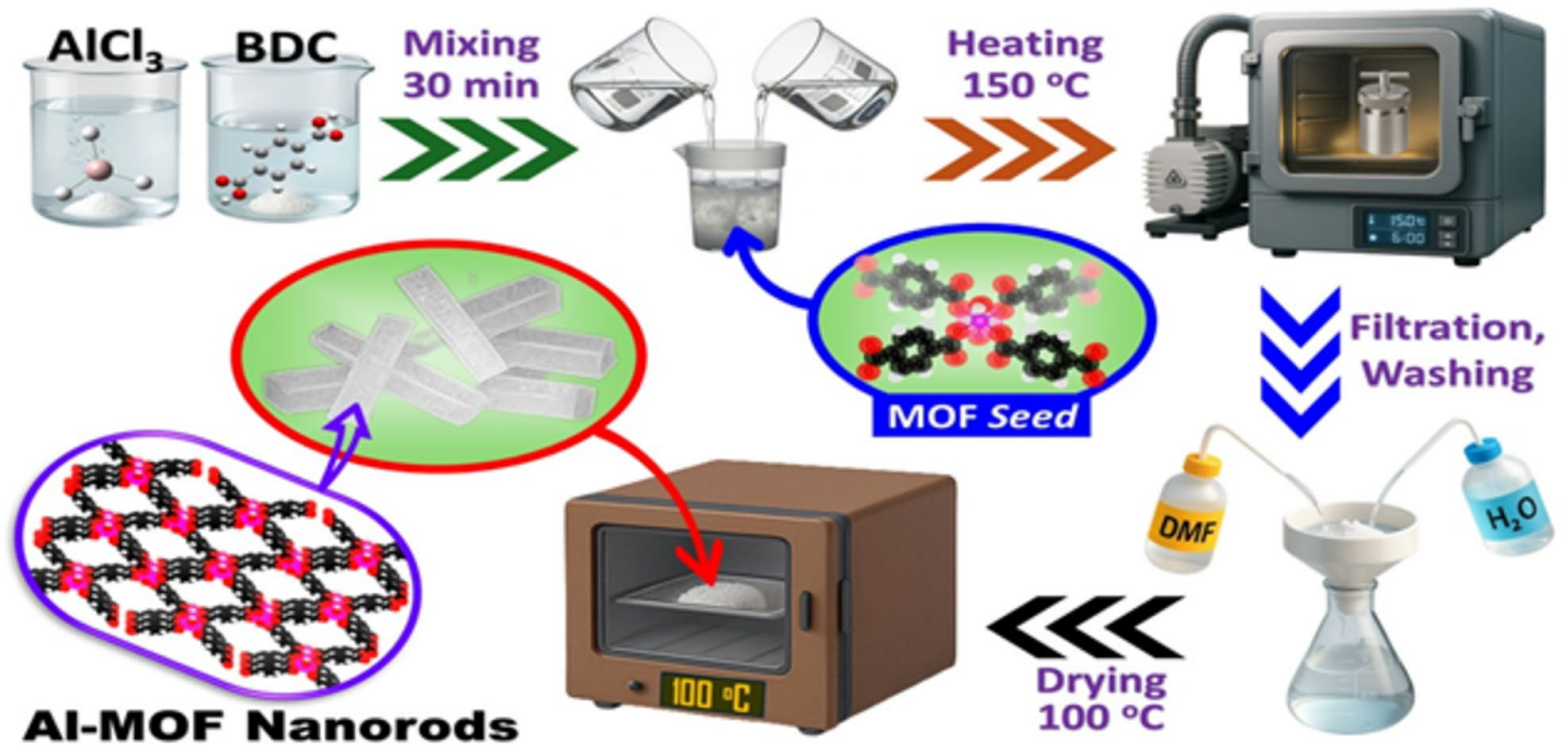
Schematic diagram for the MIL-53(Al) preparation.

Aluminium-based metal–organic framework (MIL-53(Al)) were synthesized via a modified hydrothermal route^50^, optimized for subsequent incorporation into cementitious systems. First, 4.6 g (19.05 mmol) of AlCl_3_.6H_2_O was dissolved in 90 mL deionized water (Solution A), while separately 3.2 g (19.20 mmol) of 1,4-benzenedicarboxylic acid (H_2_BDC) was dissolved in 90 mL N, N-dimethylformamide (DMF) (Solution B). Under vigorous stirring (600 rpm), Solution B was added dropwise to Solution A. After complete mixing, 1.5 mL of glacial acetic acid was introduced as a modulator, and the reaction mixture was stirred for 30 min at room temperature. The homogeneous mixture was then transferred to a 250 mL Teflon-lined autoclave and heated at 150 °C for 36 h to promote crystallization of the MIL-53(Al) framework. Upon cooling to ambient temperature, the opaque white precipitate was collected by vacuum filtration and washed twice with DMF to remove unreacted H_2_BDC, then washed three times with deionized water to eliminate residual aluminium salts. To exchange and remove occluded DMF, the solid was refluxed in methanol for 4 h, followed by filtration and overnight drying at 100 °C. Finally, the activated MIL-53(Al) was obtained by heating at 150 °C under vacuum for 6 h before characterization and blending with cement paste. Figure 1 shows a schematic diagram for the preparation process.

To investigate the synthesis of MIL-53(Al), the purity was identified using X-ray diffraction (XRD/Bruker/model-D8-Discover) via identifying the phase-composition and degree of crystallinity and structural integrity. The morphology and hierarchical structure were analyzed using a field-emission scanning-electron microscope (FE-SEM/Zeiss/model-Leo-Supra55) and high-resolution transmission electron microscopy (HR-TEM/JEOL/model-GEM-1010). The texture parameters (pore-size distribution) were evaluated via the Brunauer-Emmett-Teller model (BET) and the Barrett-Joyner-Halenda model (BJH) using an automatic surface-area and pore-size analyzer (N_2_-adsorption-desorption/MICROTRAC/model-BELSORP^®^MINI X).

### Cement specimen preparation

In this study, five mixes were fabricated, as represented in Table 2. The control specimen (coded: R) was prepared by mixing OPC and water in a mechanical mixer for 5 min. The water/cement (W/C) ratio was 0.27. For the other four mixes (coded: R-0.1MOF, R-0.3MOF, R-0.5MOF and R-1MOF), they are modified with 0.1, 0.3, 0.5 and 1 wt% MIL-53(Al), respectively. In these specimens, the W/C ratio was maintained at 0.27. The MIL-53(Al) was dispersed in the mixing water using an ultrasonic homogenizer (Labtron/model-LUHS-A12) for 15 min. The obtained solution was then mixed with OPC, as illustrated above.

**Table 2:** Mix design of the prepared pastes (mass, %).

Composite	OPC %	MIL-53(Al) %	W/C ratio
R	100	-	0.27
R-0.1MOF	100	0.1	0.27
R-0.3MOF	100	0.3	0.27
R-0.5MOF	100	0.5	0.27
R-1MOF	100	1	0.27

### Testing

The fresh properties of the prepared cementitious materials were evaluated by measuring the workability and setting time. The workability was determined using the mini-slump test (Abram cone). The setting time was measured using the Vicat apparatus (ASTM C191-19^51^). The mini-slump test was conducted at a high W/C ratio of 0.45, whereas the pastes used for setting time and compressive strength tests were prepared at a W/C ratio of 0.27, as illustrated in the mix design. This change was implemented because the low W/C mixtures showed very limited spreading, making the mini-slump test less effective in detecting the impact of MIL-53(Al) dosage. Accordingly, to evaluate distinguishable and reliable workability, the W/C ratio was increased to 0.45 to allow a meaningful comparison between the control paste and the MIL-53(Al)-modified pastes and to produce measurable spread areas that can be monitored^52–58^. Generally, the W/C ratio can influence the dispersion of MIL-53(Al) particles; it becomes easier in the high W/C ratio. Therefore, MIL-53(Al) can be easily dispersed in the W/C ratio of 0.45 rather than 0.27. However, the qualitative effect of MIL-53(Al) wt% is expected to remain similar: Accordingly, the mini-slump test was used only as a comparative study to compare the influence of MIL-53(Al) on the workability of fresh pastes.

For the hardened properties, the fresh pastes were cast in a 1-inch cubic mold, cured for 24 h at ambient temperature (25 °C) in a relative-humidity of 98 ± 2%, then demolded and cured under tap water for 1, 3, 7 and 28-days. At each time, three samples from each specimen were tested using a compression machine (Control, Max-load of 250 kN), then average values were calculated (ASTM C109M-20b^59^. After that, the phase composition of hydration products and the microstructure of cement matrices were investigated using XRD/Malvern-PANalytical/model-X’Pert-Pro and SEM/Thermo-Scientific/model-Quatro-S connected with an X-ray analyzer (EDX), respectively.

The anti-bacterial activity of some selected specimens (R, R-0.1MOF and R-0.5MOF) was evaluated by measuring the impact of MIL-53(Al) and its dosages on the inhibition of growing two gram-positive bacteria (*Bacillus subtilis* (ATCC 6633) and *Staphylococcus aureus* (ATCC 6538)) and two gram-negative bacteria (*Klebsiella pneumoniae* (ATCC 13883) and *Salmonella typhi* (ATCC 6539)). A disc (length∗width∗thickness of 2.5*2.5*0.3 cm) from each specimen was located in a Malt agar plate (MAP) at 28 ± 1 °C containing the bacteria mentioned above. Generally, high alkalinity of cement exhibits high anti-bacterial activity, as it results in disturbing enzymatic activity and damaging bacterial cell membranes. Accordingly, prior to anti-bacterial testing, these discs were exposed to air carbonation to lower the cement matrix’s alkalinity to remove its influence and allowed for a focused study on MIL-53(Al)’s specific role in inhibiting bacterial growth. The carbonation process was conducted in air for 28-days under an ambient temperature of 25 °C and relative humidity of 45 ± 2% to simulate real curing conditions of building material used in anti-bacterial applications. After that, the discs were immersed in distilled water for 3-days with a continuous washing until the pH of the flooding water reached 8 ± 0.2. Also, pH values of the agar medium before and after contact with the discs were measured to verify that the cement discs didn’t cause an increase in the agar’s pH after incubation. The pH of agar before contact with the cement discs was found to be 7.2–7.4; after incubation, it increased to about 8.1. This demonstrates that the pH of agar surrounding discs did not show a significant increase in alkalinity, indicating that the anti-bacterial activity will be attributed to the inclusion of MIL-53(Al) in the cement matrix, not dominated by the alkalinity of discs. According to the National-Committee for Clinical-Laboratory Standards (CLSI), the MAP was prepared by autoclaving 30 ml of Sabroud-dextrose-agar medium at 121 °C/15Ibs, which solidified with 0.1 ml of bacteria^60^. According to ASTM D4300-01, the anti-bacterial activity was assessed via inhibition-zone measurements around discs^61^.

## Results and discussion

### The structural characterization of MIL-53(Al)

#### XRD analysis

**Fig. 2:**
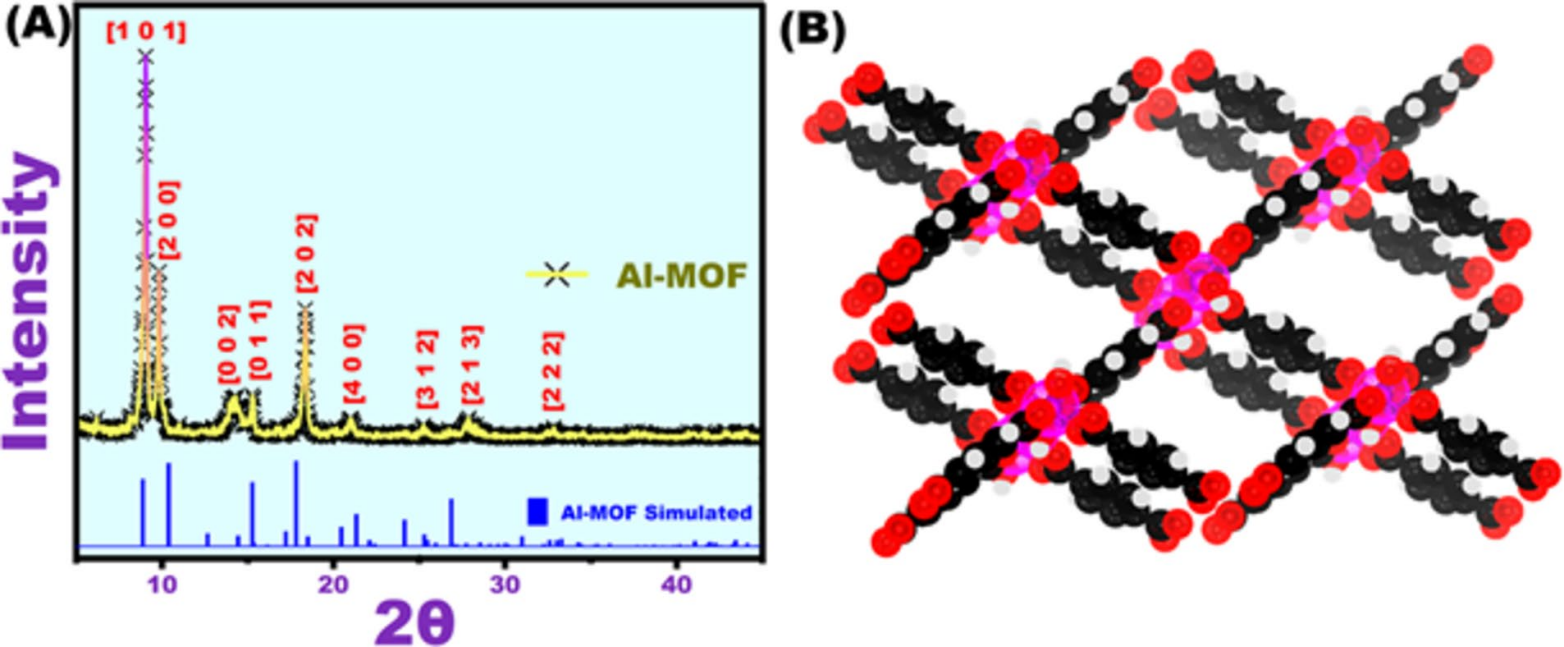
Phase identification of MIL-53(Al): (**A**) Powder X-ray diffraction pattern and (**B**) a three-dimensional representation of the crystal structure.

The structural integrity and crystalline properties of the synthesized MIL-53(Al) were comprehensively evaluated using powder X-ray diffraction (PXRD) analysis (Fig. 2). Figure 2a shows the X-ray diffraction pattern of the synthesized MIL-53(Al) (yellow) compared with the simulated MIL-53(Al) structure (blue) (Reference code: 96-430-5222), showing characteristic peaks with its Miller indices indicated. This confirms the successful formation of a highly ordered MIL-53(Al) framework suitable for cement reinforcement applications. The pristine MIL-53(Al) (Fig. 2a) exhibits exceptional crystallinity, as evidenced by sharp, well-defined diffraction peaks at 2Ө values of 9.05, 9.83,14.3, 15.26, 18.35, 20.99, 25.25, 27.89 and 32.84°, which closely match the simulated MIL-53(Al) structure reported in the literature^62^. The absence of additional crystalline phases or amorphous content confirms the phase purity of the MIL-53(Al), which is crucial for predictable performance in cement composites. The predominant reflections at 2Ө = 9.05, 9.83 and 18.35° correspond to the [101], [200] and [202] reflections, and crystallographic planes, respectively, confirming the formation of an orthorhombic unit cell (Fig. 2b) with space group Pnma and lattice parameters of a = 17.12 Å, b = 6.68 Å, and c = 12.36 Å ^50^. In Fig. 2b, aluminum nodes, oxygen, carbon and hydrogen atoms have a color code pink, red, black and white, respectively. Also. It illustrates the porous architecture suitable for cement reinforcement applications.

#### SEM and HR-TEM Analysis

**Fig. 3:**
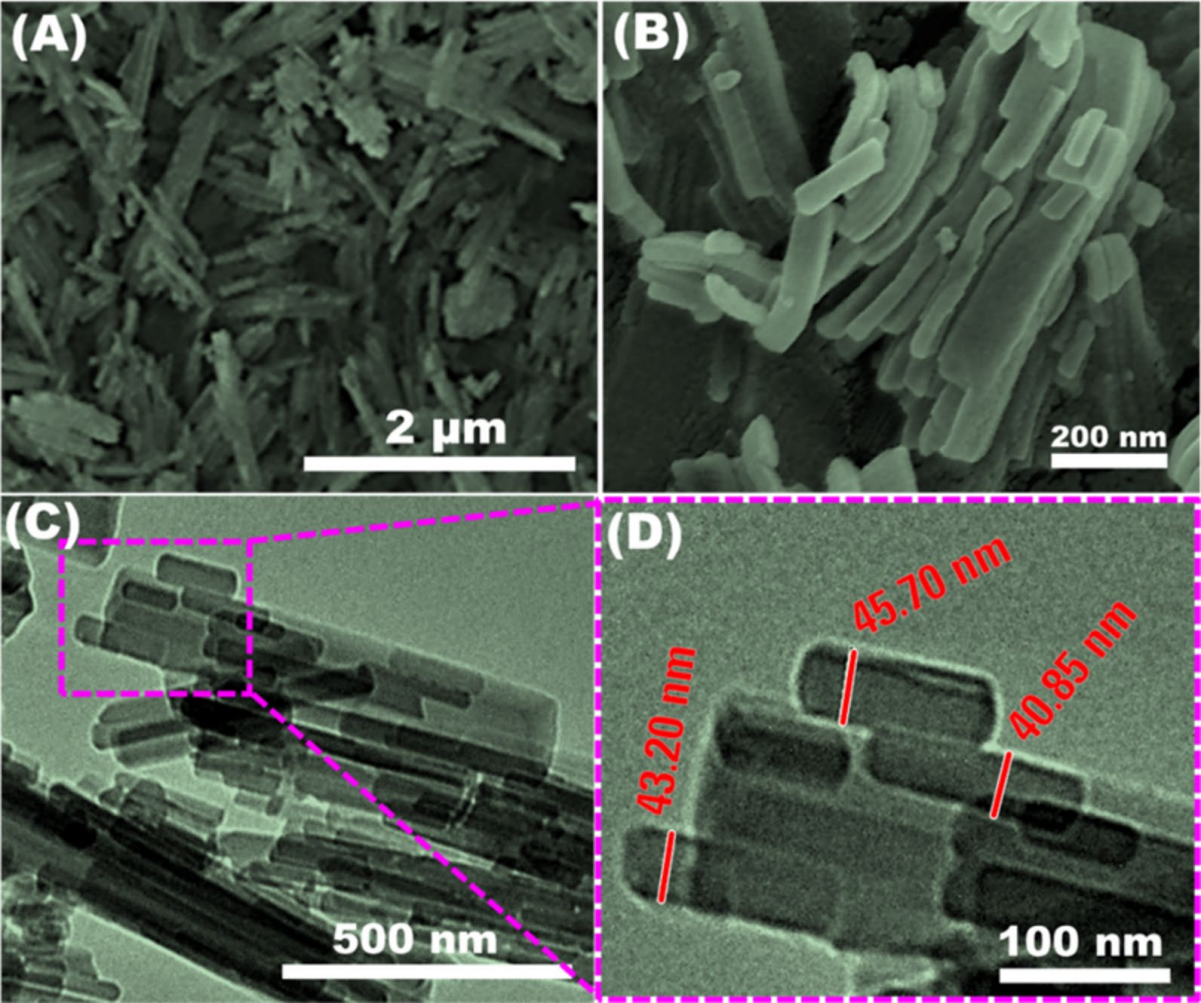
Morphological characterization of MIL-53(Al): (**A**,** B**) FESEM images (**C**,** D**) HRTEM images.

The morphological evolution and hierarchical structural development of the MIL-53(Al) were comprehensively analyzed using FESEM and HR-TEM at various magnification levels, as depicted in Fig. 3. The hydrothermal synthesis process successfully yielded well-defined rod-shaped MIL-53(Al) nanocrystals with nanometer-scale dimensions, demonstrating the transformation from micrometer-sized raw materials into highly ordered hierarchical nanostructures^50,63^. Comprehensive microscopic analysis reveals that the MIL-53(Al) exhibits a uniform coral-like cluster morphology composed of irregularly elongated rod-like structures with excellent size distribution and dispersion characteristics. The hierarchical rod-shaped clustering observed in the SEM images, attributed to hydrogen bonding interactions between carboxyl groups of the organic linkers, creates an interconnected network structure. As observed in (Fig. 3a, b), the individual nanorods display smooth surfaces with well-defined edges, multiple fracture planes, and distinct hexagonal cross-sections, with crystal lengths approximately 500 nm and diameters averaging 43 nm as confirmed by HR-TEM analysis (Fig. 3c, d). This refers to the hierarchical nanorod architecture having a high ratio (length-to-diameter ratio ~ 11:1).

#### Surface analysis

**Fig. 4:**
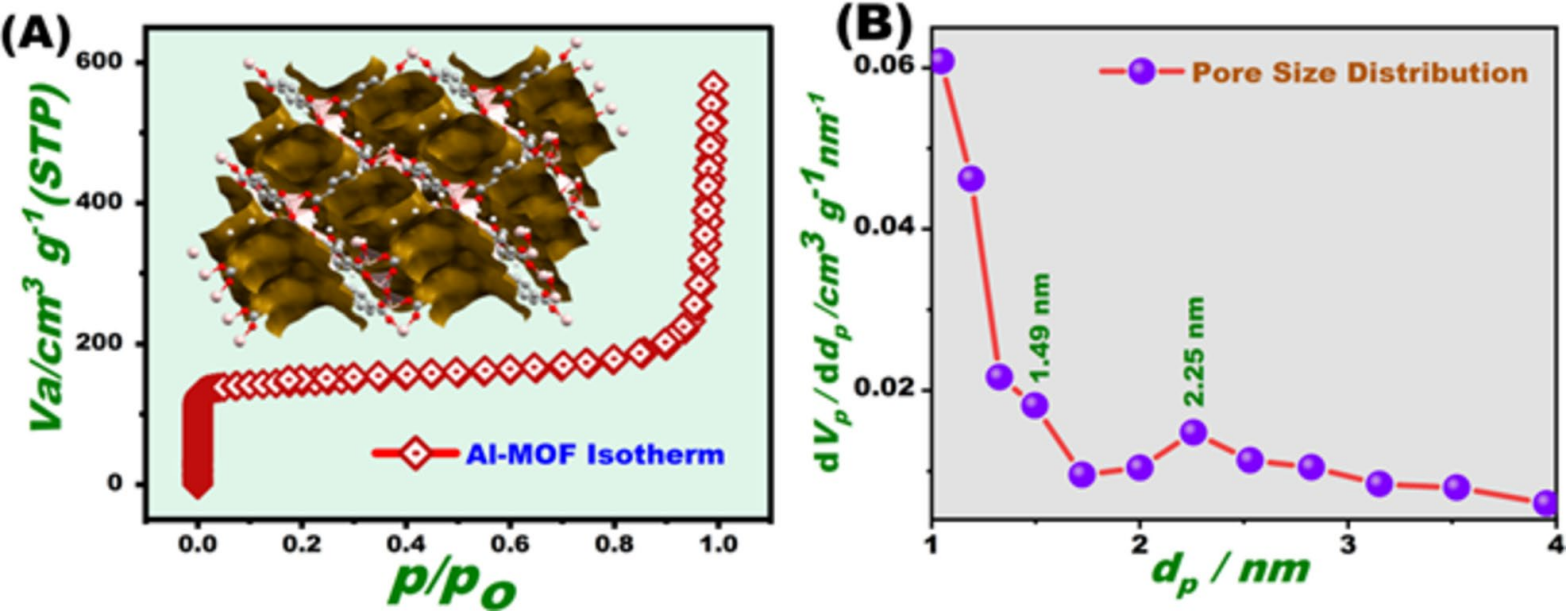
Texture characteristics of MIL-53(Al): (A) Nitrogen adsorption–desorption isotherm at 77 K and (B) Differential pore size distribution derived by the BJH method.

The nitrogen adsorption–desorption isotherm of the MIL-53(Al) (Fig. 4a) exhibits a combined Type I/IV profile, indicating a hierarchical pore network comprising both micropores and mesopores. A steep uptake at low relative pressures (p/p_o_ < 0.1) confirms a dominant microporous framework, while a secondary uptake and hysteresis loop at p/p_o_ > 0.4 reflects mesopore formation with pore-neck structures typical of MIL-53(Al)^50^. It was found that the Brunauer–Emmett–Teller surface area (BET) of it is 455 m^2^/g and the total pore volume of 0.87 cm^3^/g. The differential pore size distribution (Fig. 4b) reveals two primary pore diameters at 1.49 nm and 2.25 nm, corresponding to intrinsic micropore windows and mesoporous cavities, respectively, and an average mesopore diameter of 7.6 nm.

### Fresh properties

**Fig. 5:**
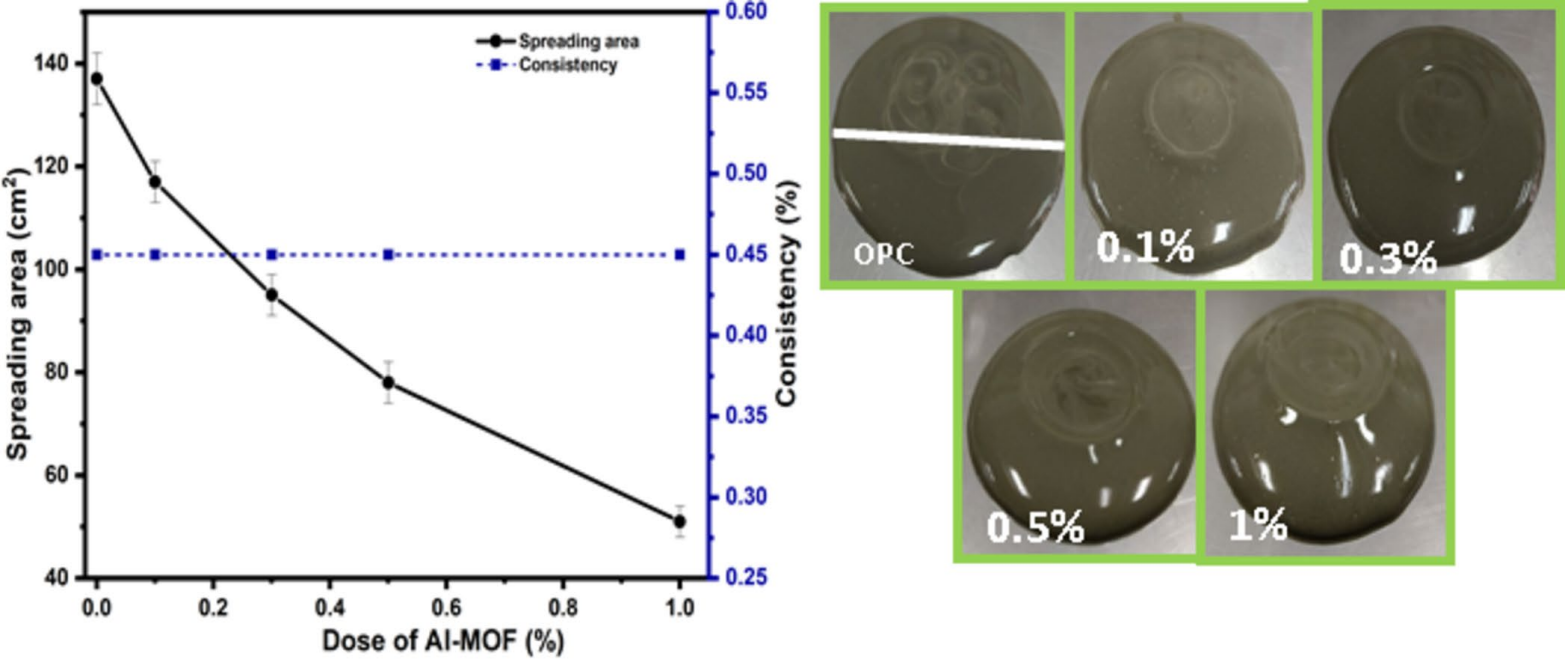
Impact of MIL-53(Al) addition on the flowability of OPC fresh pastes.

**Fig. 6:**
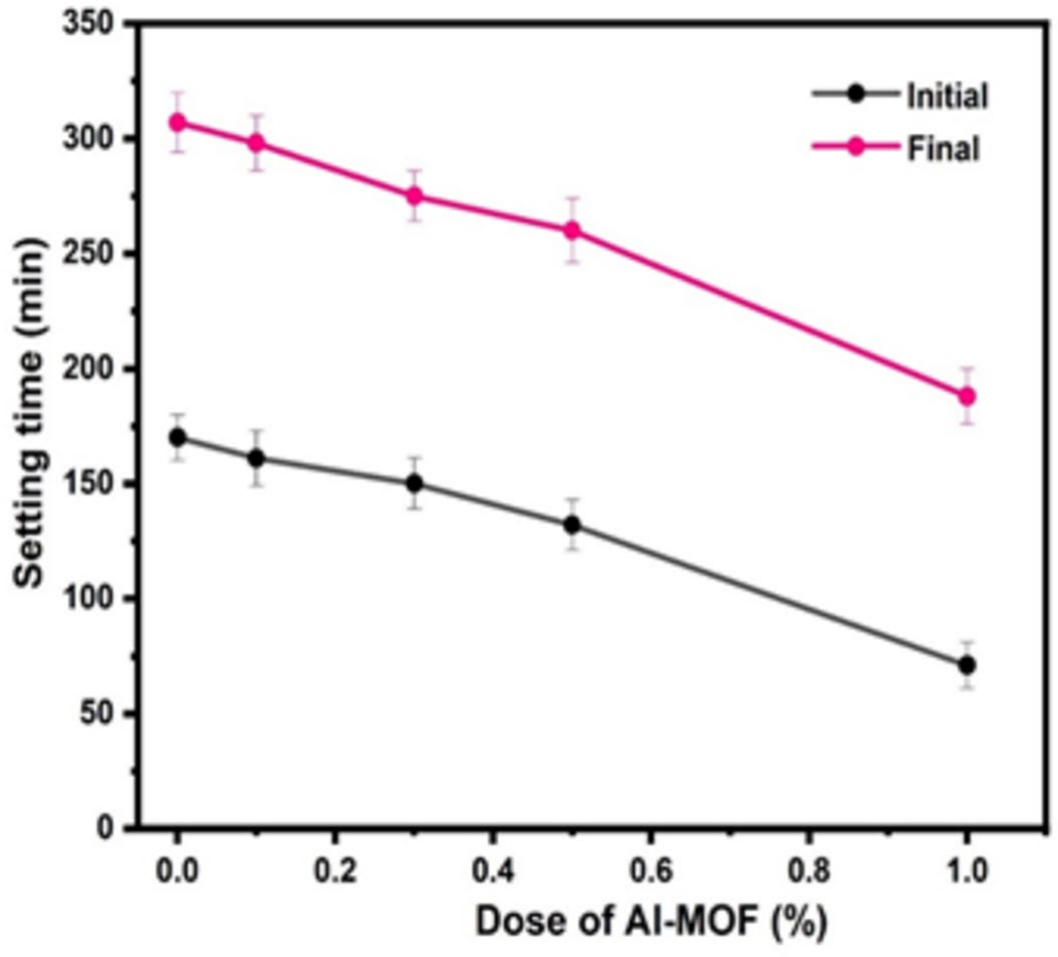
Impact of MIL-53(Al) addition on the setting times of OPC pastes.

Generally, the workability and setting time of cementitious materials are considered as main factors that identify the time required for transportation, placement and compaction^64,65^. Accordingly, the impact of 0.1, 0.3, 0.5 and 1 wt% MIL-53(Al) on the workability and initial/final-setting time (I/F-ST) were measured as represented in Figs. 5 and 6. Regarding the workability (Fig. 5), a significant reduction in the workability (spread-area value^66^ is detected with increasing the dosages of MIL-53(Al); it decreases by 14.6, 30.7, 43.1 and 62.8% for R-0.1MOF, R-0.3MOF, R-0.5MOF and R-1MOF, respectively, concerning the R specimen (control). These findings are consistent with other research; Yuan et al. ^27^ and El-Mir et al. ^67^ proved that certain MOF types, including MIL-68(Al), UiO-66, and ZIF-67, reduce workability. The slump losses after inclusion of MIL-53(Al) in the cement matrix may be associated with (i) its high surface area compared to OPC (455 and 0.38 m^2^/g, respectively), which increases the water demand^67,68^; (ii) its coral-like cluster morphology, which is consists of elongated rod-like increases the friction and interlocking between cement grains and hence hinders the flowability^69,70^; (iii) its filling impact that transforms the cementitious matrix from a mobile to a stationary state, which also prevents the flowability^71^.

For the setting time (Fig. 6), it is noticed that MIL-53(Al) has an acceleration impact. The IST for R-0.1MOF, R-0.3MOF, R-0.5MOF and R-1MOF were shortened by about 9, 20, 38 and 99 min, while the FST shortened by 9, 32, 47 and 119 min with respect to the R specimen, respectively. Interestingly, noting that setting times for all cement pastes fall between the acceptable range of cementitious materials (45 and 375 min, ASTM C150/C150M-20^72^). The high surface area of MIL-53(Al), which increases the capability to absorb mixing water, as well as its filling effect increases the stiffness of fresh pastes, leading to shortening of setting time^65,71^. Furthermore, the ability of MIL-53(Al) to act as nucleation seeds accelerates the hydration process to form more strength-giving phases, such as tobermorite (calcium-silicate-hydrate, CSH), strätlingite (calcium-alumino-silicate-hydrate, CASH), and hydrocalumite and/or hydrogarnet (calcium-aluminate-hydrate, CAH)^70,73^. The precipitation of strength-giving phases on MIL-53(Al), instead of unreacted cement particles, enables water to reach them, promoting the hydration process^74,75^.

### Compressive strength

**Fig. 7:**
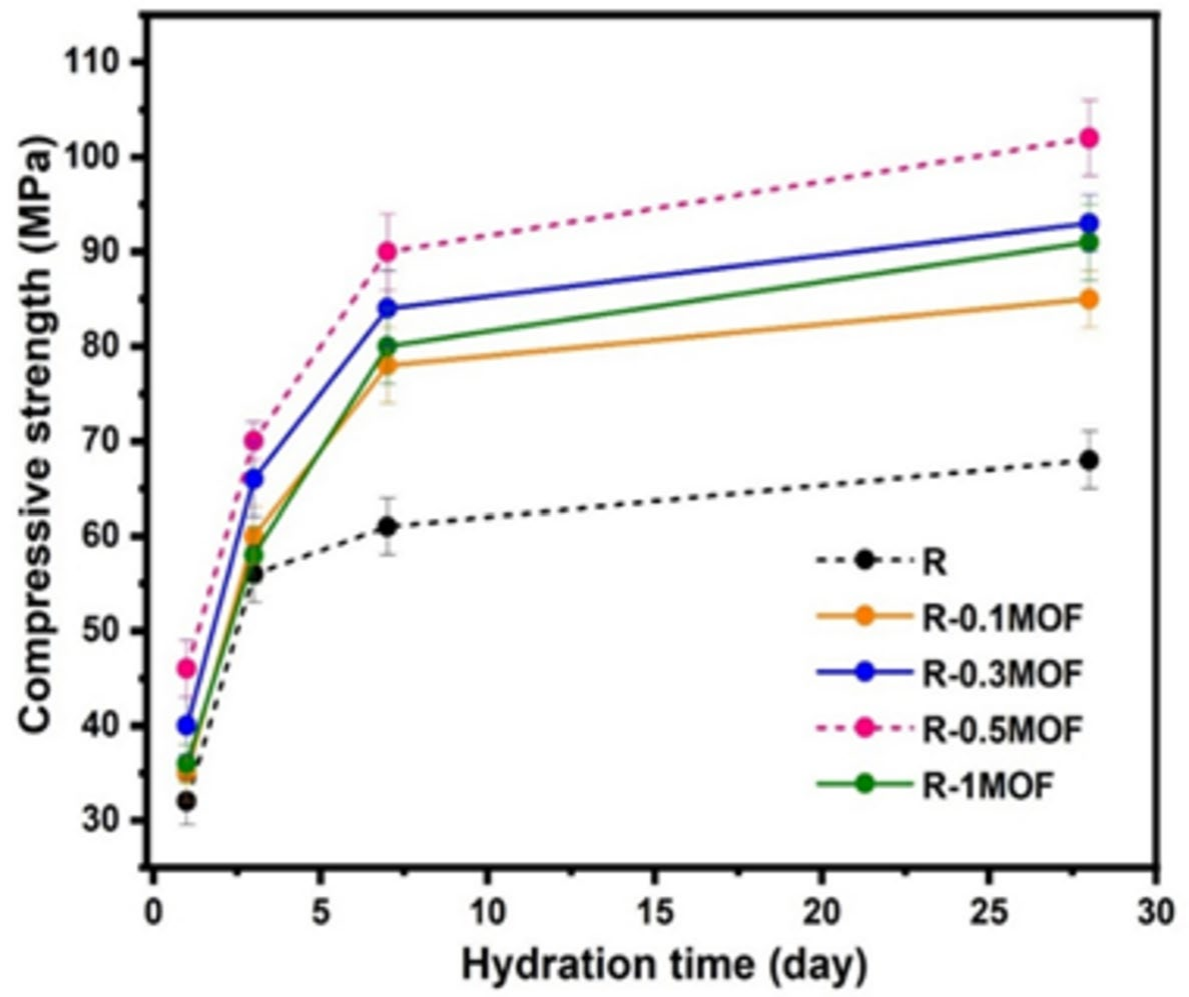
Compressive strength values with hydration time for OPC pastes modified with different doses of MIL-53(Al).

The compressive strength values of the cementitious materials are used to evaluate their quality for construction applications. Therefore, the compressive strength values of the specimens modified with different dosages of MIL-53(Al) were compared with the control specimen (0 wt% MIL-53(Al)) to evaluate its impact on the mechanical performance, as in Fig. 7. Generally, a significant increase in compressive strength is observed with curing time; they are enhanced by 112.5, 142.8, 132.5, 121.7, and 152.7% from 1 to 28 days for R, R-0.1MOF, R-0.3MOF, R-0.5MOF and R-1MOF, respectively. Accumulation of strength-giving-phases, such as tobermorite, strätlingite and hydrocalumite and/or hydrogarnet) that result of the hydration of the OPC with time fills the open pores, increasing the gel/space ratio, thus enhancing the compressive strength^76,77^.

**Fig. 8:**
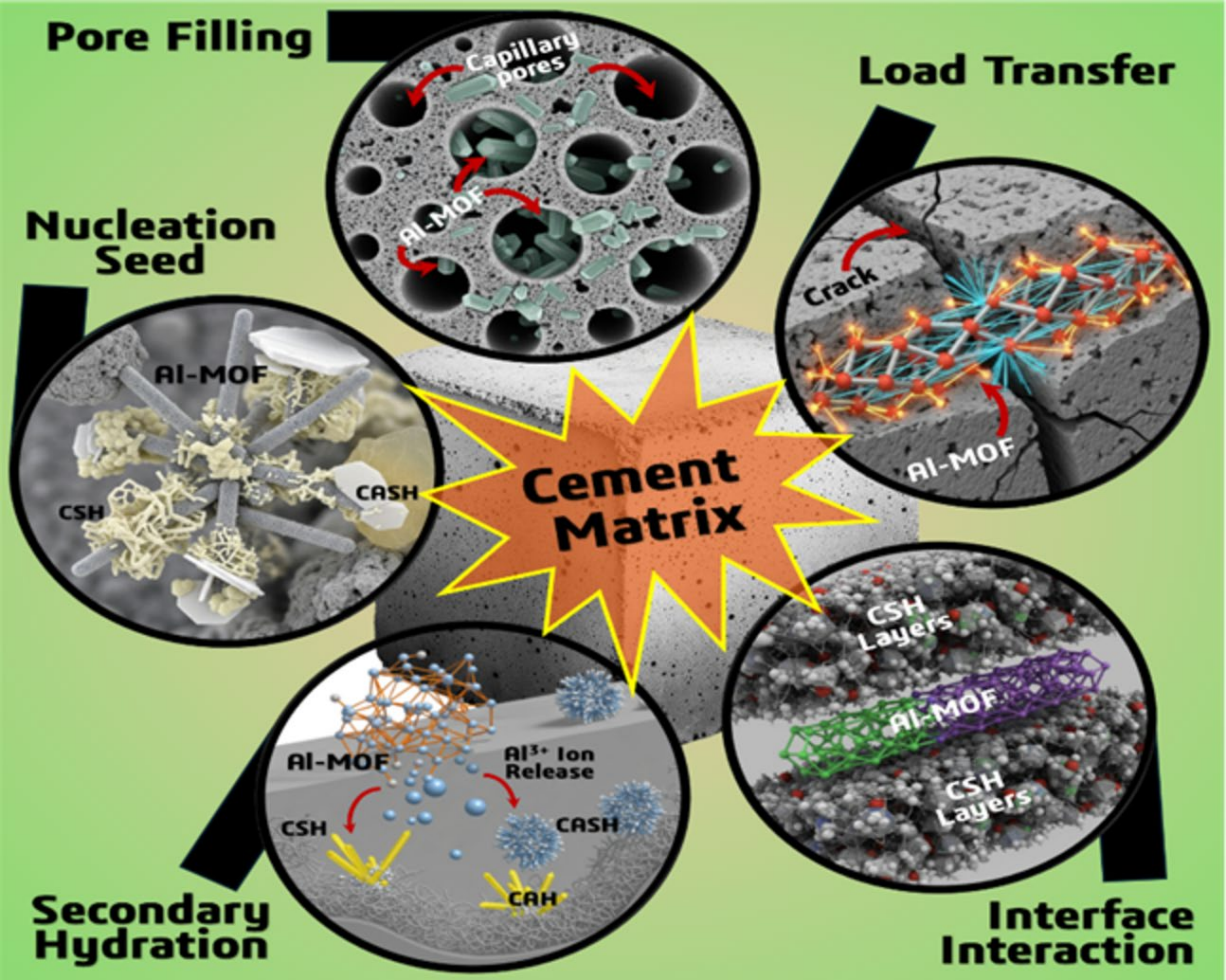
Schematic diagram showing the role of MIL-53(Al) in enhancing mechanical performance.

Interestingly, noting that the specimens modified with MIL-53(Al) achieve a considerable enhancement in the compressive strength values at early and later ages with respect to the control specimen. At 1-day, compressive strengths are enhanced by 9.4, 25.0, 43.8 and 12.5%, while at 28-days, they are enhanced by 25, 36.8, 50.0 and 33.8% for specimens modified with 0.1, 0.3, 0.5 and 1 wt% MIL-53(Al), respectively. These results show that the compressive strength is enhanced by increasing the dosage up to 0.5 wt%; beyond this (at 1 wt%) the strength decreases but remains higher than the control specimen (0 wt%). The enhancement in the compressive strength may be attributed to different mechanisms: (i) filler effect: Because of the high fineness of MIL-53(Al), it can occupy capillary pores, as well as the gel/interlayer pores created when hydration products (CSH, CAH and CASH) form between cement particles. Increasing the dosage of MIL-53(Al) to 0.5 wt% results in a denser internal structure of the hardened specimens by refining the pores, which enhances their strength^78,79^; (ii) nucleation seeds: Also, due to the high surface area of MIL-53(Al), in addition to its micro/meso-pores texture characteristics, it can act as active seeds for precipitation hydration products, promoting the dissolution/hydration of unreacted cement particles and thus improving microstructure development^80^; (iii) the formation of secondary hydration products: MIL-53(Al) facilitates the gradual availability of Al^3+^ ions of (cluster/chain of Al^3+^ ions) at a highly alkaline medium^81^, which can react with portlandite (Ca(OH)_2_, CH) forming extra amount from strength-giving-phases (CAH and CASH)^70^. Gecgel et al. ^82^ and Arunkumar et al. ^83^ reported that MOFs are considered as metal reservoirs; at the same time, it has a high ability for the gradual release of these metal ions, making them suitable for different applications; (iv) interface interaction and load transfer: the carboxylic group in the chemical structure of MIL-53(Al) can form a covalent bond with the surface of hydration products, additionally, its elongated nano rod-like structures leads to load transfer between MIL-53(Al) and the cement paste, as well as prevent crack propagation, improving the mechanical characteristics^80,84–86^. Figure 8 shows a schematic diagram that describes the role of MIL-53(Al) in enhancing compressive strength. Finally, the reduction in compressive strength with increasing the dosage of MIL 53(Al) up to 1 wt% can be attributed to its agglomeration^36,87^. This agglomeration negatively impacts mechanical properties via (i) the formation of weak zones due to its uneven distribution in the cement matrix. Around the formed clusters, the packing of the matrix becomes less homogeneous, and hydration products are obtained less uniformly^27,88^; (ii) reducing the external surface area diminishes the MIL-53(Al)’s effectiveness as nucleation sites^89,90^; and (iii) weakening the interfacial transition region between the MIL-53(Al) particles and the hydration products. Rather than creating a continuous cementitious matrix, the system may consist of particle-rich regions encased by less dense hydration products^91^. Under load, these regions can initiate microcracks earlier than in the well-dispersed system^92,93^. Also, owing to the micro/meso-pores structure of MIL-53(Al), it can adsorb a higher amount of mixing water, causing (i) a decrease in the available water needed for cement hydration, resulting in fewer hydration products^94,95^; and (ii) reducing the workability, as demonstrated above, the inclusion of 1 wt% MIL-53(Al) reduced the workability of the cement paste by 62.8%. Poor workability makes compaction difficult, resulting in trapped air voids^96,97^.

### Phase composition

**Fig. 9:**
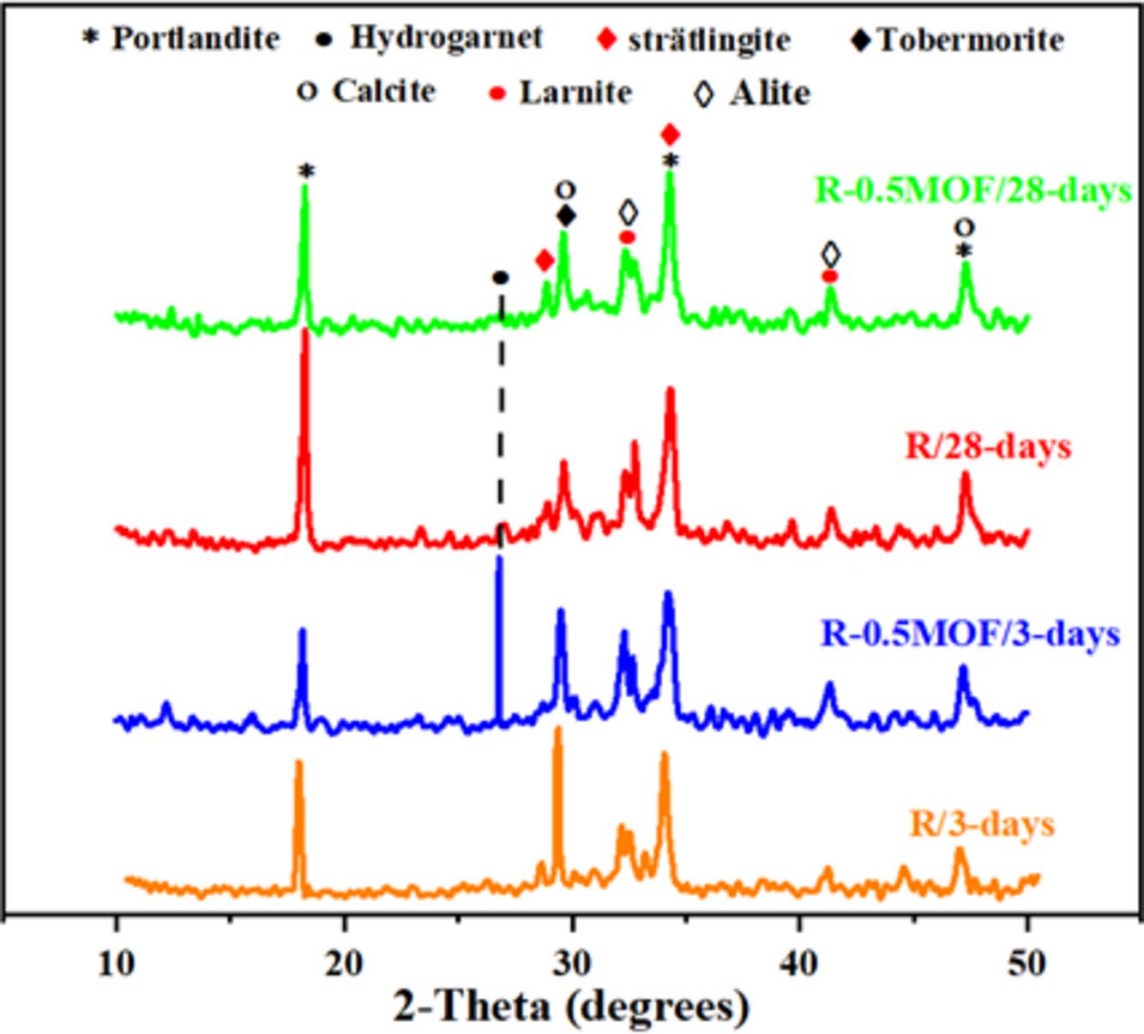
Phase composition analysis of R and R-0.5MOF specimens at 3 and 28-days.

The phase composition of the hardened specimens (R and R-0.5MOF) at 3 and 28-days was examined using XRD to evaluate the impact of MIL-53(Al) on the hydration process of OPC, as represented in Fig. 9. The XRD-pattern of the R/3-days specimen shows several distinguishable peaks that may be associated with unreacted OPC phases and formed hydration products. The unreacted phases appeared at 2Ɵ= 32.24 and 41.47°, which may be attributed mainly to residual calcium silicate clinker phases, including larnite/belite (Ca_2_SiO_4_, β-C_2_S, PDF# 96-901-7425) with possible overlap from alite phase (Ca_3_SiO_5_, C_3_S, PDF# 96-154-0706), Because the principal reflections of β-C_2_S and C_3_S are close to each other in OPC systems, these peaks cannot be assigned to a single clinker phase with complete certainty. Nevertheless, their persistence suggests that the hydration process was still incomplete at 3-days, which is expected due to the gradual hydration of residual silicate phases, especially belite^98^.

Regarding the hydration products, the presence of peaks at 2Ɵ= 18.12, 34.14 and 47.10° is consistent with the formation of portlandite (Ca(OH)_2_, CH, PDF# 96-100-0046), which can be carbonated by atmospheric CO_2_, causing the possible formation of calcite phase (CaCO_3_, CC, PDF# 96-100-0046) at 2Ɵ= 29.34 and 47.10° ^99^. The peak near 2Ɵ= 29.34° is commonly used as a major indicator of calcite. Also, the compressive strength of the R specimen at 3-days (56 MPa) can be discussed by the development of hydration products that contribute to matrix densification and strength enhancement, such as calcium-aluminosilicate-hydrate-type phases, including possible strätlingite-like phases (C_2_ASH_8_, Ca_2_Al_2.11_Si_1.11_O_16.25_H_18_, PDF# 96-900-5060) with reflections that may contribute to the region around 2Ɵ= 28.74 and 394.17°. In addition, a peak around 2Ɵ= 29.34–29.69° may be compatible with poorly tobermorite phase (CSH, Ca_2_Si_3_O_11_H_6_, 2Ɵ= 29.69°, PDF# 96-900-2247)^70,100,101^. By comparing the XRD-pattern of the R/3-days specimen with that of R-0.5MOF/3-days, the same peaks are observed with different intensities. In addition, a new peak is observed at 2Ɵ= 26.76°, which may be compatible with poorly crystalline C–S–H or tobermorite-like ordering (C_3_AH_6_, Ca_3_Al_2.8_O12H_9.6_, PDF# 96-900-5160). The reduction in the intensity of the portlandite peaks and the appearance of the hydrogarnet peak suggest the ability of MIL-53(Al) to influence the aluminate-related hydration reactions, possibly through the gradual availability of Al-containing species and/or through providing additional nucleation sites for hydrate precipitation^82^. The portlandite may react with Al^3+^ ions, forming hydrogarnet and an extra amount of strätlingite as secondary strength-giving-phases^83,102^. This discusses the enhancement in the compressive strength after the inclusion of 0.5 wt% MIL-53(Al) in the cement matrix. It increases from 56 MPa (R/3days) to 70 MPa (R-0.5MOF/3-days).

On the other hand, the XRD pattern of the R specimen at 28 days (R/28-days) demonstrates a significant increase in the peaks of portlandite, indicating progression in the hydration process, and thus increasing the compressive strength values (68 MPa)^70^. However, the XRD pattern for R-0.5MOF/28-days shows the lowest intensity of peaks for unreacted phases (larnite and alite). This supports the role of MIL-53(Al) in acting as a nucleation seed for the formation of hydration products on its surface, promoting the hydration of unreacted particles^80,103^. The high surface area and porous framework of MIL-53(Al) may provide favorable sites for the precipitation of C-S-H/C-A-S-H-type products, thereby accelerating cement hydration and reducing the amount of residual clinker phases. Furthermore, the possible Al contribution from MIL-53(Al), together with its nucleation effect, may promote the formation of additional alumina-bearing hydrates such as hydrogarnet-like and strätlingite-like phases. This discussed the highest compressive strength values achieved by R-0.5MOF/28-days (102 MPa).

### Microstructure

**Fig. 10:**
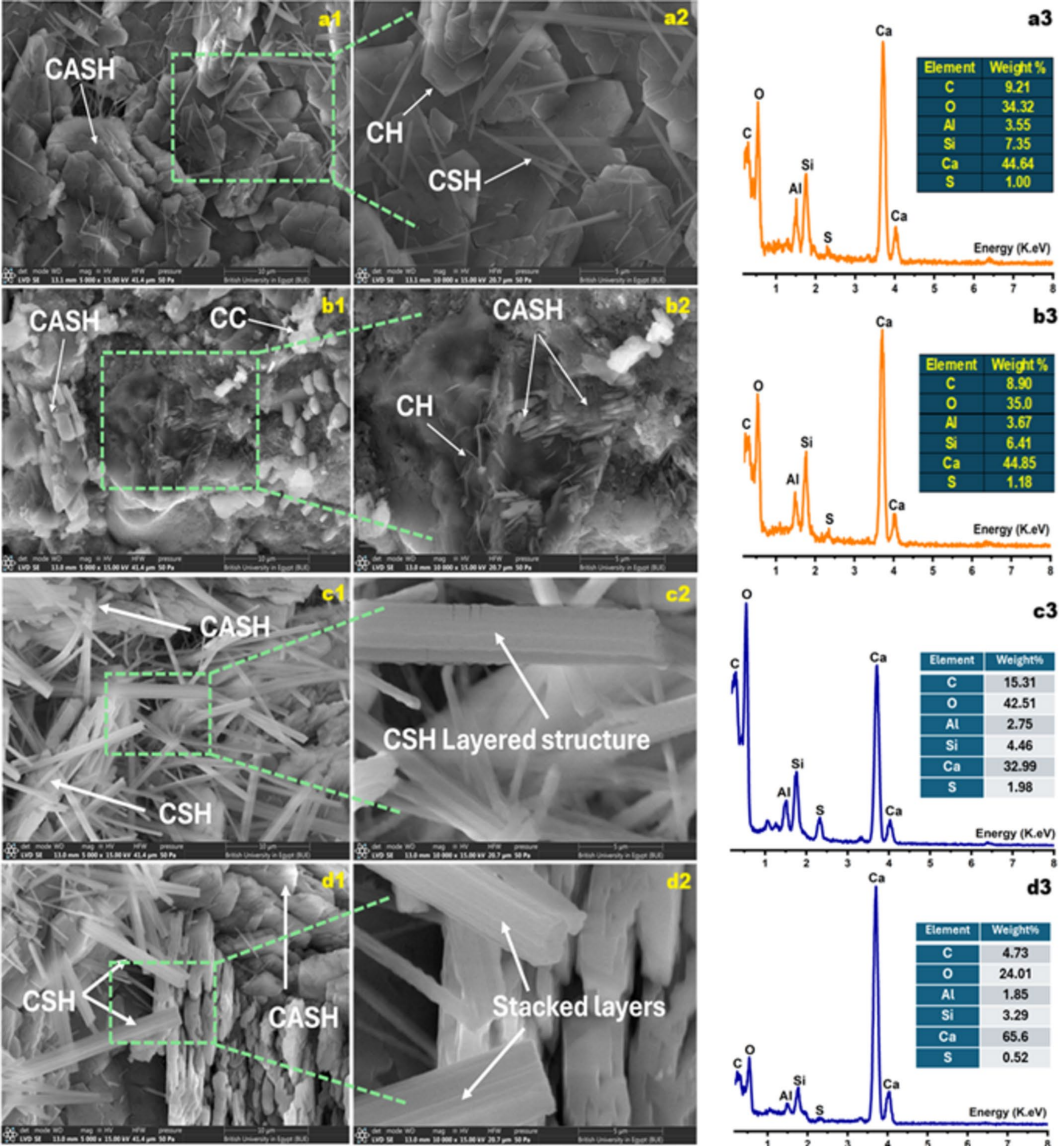
SEM/EDX images of: R (a1-b3) and R-0.5MOF (c1-d3) hardened pastes at 3 and 28-days of curing.

Figure 10 illustrates the SEM/EDX microstructural and elemental analyses of both the reference cement paste (R) and the paste modified with 0.5 wt% (MIL-53(Al), designated as R-0.5MOF, after 3 and 28 days of ambient curing in tap water at room temperature. SEM images of the reference paste at 3 days (Fig. 10a1, a2) reveal a microstructure dominated by needles of calcium silicate hydrate (C-S-H) crystals^104^, interlaced with layered and stacked calcium aluminate silicate hydrate (C-A-S-H) formations^105,106^. Additionally, hexagonal portlandite (CH) crystals^107^ were prominently observed, indicating active early-stage hydration. By 28 days (Fig. 10b1, b2), the reference paste exhibited a significantly denser and more compact microstructure, primarily attributed to the accumulation of stacked hexagonal plates of strätlingite (C_2_ASH_8_)^108^. This densification reflects progressive hydration and suggests improved mechanical integrity over time.

The incorporation of 0.5 wt% MIL-53(Al) into the cement matrix significantly accelerated the hydration process^23^, particularly at early curing stages. At 3 days (Fig. 10c1, c2), the modified paste exhibited a pronounced increase in the formation of calcium silicate hydrate networks and a modest presence of calcium aluminate silicate hydrate (C-A-S-H) plates^109–111^, with a notable absence of portlandite (CH), suggesting a shift in the hydration pathway. This early enhancement in hydrate formation indicates that MIL-53(Al) acts as an effective nucleation site, promoting rapid phase development and reducing the formation of less desirable crystalline phases^112,113^. As curing progressed to 28 days (Fig. 10d1, d2), the microstructure evolved into a more refined and densely packed configuration. SEM observations revealed the presence of layered C-S-H structures and short rod-like formations, accompanied by a substantial accumulation of stacked C-A-S-H phases. In addition to its high surface area, which accelerates hydration by providing abundant nucleation sites, MIL-53(Al) may contribute to microstructural refinement through facilitating the gradual availability of Al^3+^ ions^82,114,115^ into the cement pore solution. These mobile aluminum ions integrate into the growing CAHs and C-A-S-H gel network, promoting the formation of more cross-linked and stable hydrates^116,117^. This dual mechanism, physical enhancement via surface area and chemical modification via ion diffusion, collectively suppresses portlandite (CH) formation and fosters the development of a denser, more durable cement matrix^27^.

Figure 10a3, b3, c3, d3 presents the EDX spectra of both reference (R) and R-0.5MOF cement pastes at 3 and 28 days of hydration. At 3 days, elemental analysis revealed Ca/Si and Al/Si ratios of 5.10 and 0.59 for the R paste, while the R-0.5MOF paste exhibited elevated values of 5.18 and 0.64, respectively (Fig. 10a3, c3). Based on atomic percentages, the R paste exhibited Ca/Si and Al/Si ratios of 5.10 and 0.59 at 3 days, respectively, whereas the R-0.5MOF paste showed slightly higher values of 5.18 and 0.64 (Fig. 10a3 and c3). These results suggest that the incorporation of 0.5 wt% MIL-53(Al) influenced the composition of the hydration products, likely through its nucleation effect, which can promote the dissolution of calcium silicate phases (C₃S and β-C₂S), resulting in increased calcium ion release and the formation of calcium-rich C-S-H gel^118^. Simultaneously, the MIL-53(Al)’s porous framework facilitates the gradual availability of Al^3+^ ions into the pore solution, which not only promotes the formation of ettringite and AFm phases but also enables aluminum substitution for silicon within the C-S-H structure^119^. This substitution favors the development of calcium aluminate silicate hydrates (CASH), contributing to the observed rise in the Al/Si ratio^120^. After 28 days, the R-0.5MOF paste exhibited a higher Ca/Si ratio (13.96) than the reference paste (4.90), indicating that MIL-53(Al) influenced the composition and evolution of the hydration products, promoting the formation of calcium-rich hydrates, which developed the mechanical performance to 102 MPa.

These findings agree with the work of Zheng et al.^121^ who demonstrated that microstructural densification and defect reduction are key factors governing the mechanical performance of cementitious materials. Although the reinforcement mechanism differs, the incorporation of MIL-53(Al) likewise resulted in a denser microstructure and refined pore network, which can explain the enhancement in compressive strength observed at 28 days. Previous studies^122–124^ on aluminosilicate-based stabilization systems have shown that variations in CaO/Al_2_O_3_ and SiO_2_/Al_2_O3 ratios significantly influence the formation of binding phases and the resulting mechanical properties. Furthermore, the mineralogical composition of the precursor materials has been reported to affect the development of reaction products and microstructural stability. In the present study, MIL-53(Al) likely contributed to modifying the local Ca-Al-Si environment, favoring the formation of additional CASH-type phases and a denser microstructure. Moreover, the rod-like morphology observed for some hydration products may contribute to improved particle interlocking and load transfer, consistent with reports demonstrating that particle shape and aspect ratio can influence packing behavior and mechanical response. These combined chemical and morphological effects provide additional support for the enhanced strength observed in the MOF-modified cement paste.

### Antibacterial activity

The bacteria *Bacillus subtilis* (ATCC 6633 – Gram-positive, rod-shaped), *Staphylococcus aureus* (ATCC 6538 – Gram-positive, spherical), *Klebsiella pneumoniae* (ATCC 13883 – Gram-negative, rod-shaped), and *Salmonella typhi* (ATCC 6539 – Gram-negative, rod-shaped) can negatively affect both human health and construction materials. These microbes are known to cause serious infections such as food poisoning, pneumonia, and skin diseases. Some, like S. aureus, are especially dangerous due to their resistance to many antibiotics^125^. In building environments, these bacteria can grow on cement surfaces and slowly damage them. They produce acids and biofilms that weaken concrete, reduce its strength, and cause corrosion in steel reinforcements. This leads to faster deterioration of buildings, especially in moist or polluted areas^45,126^.

**Fig. 11:**
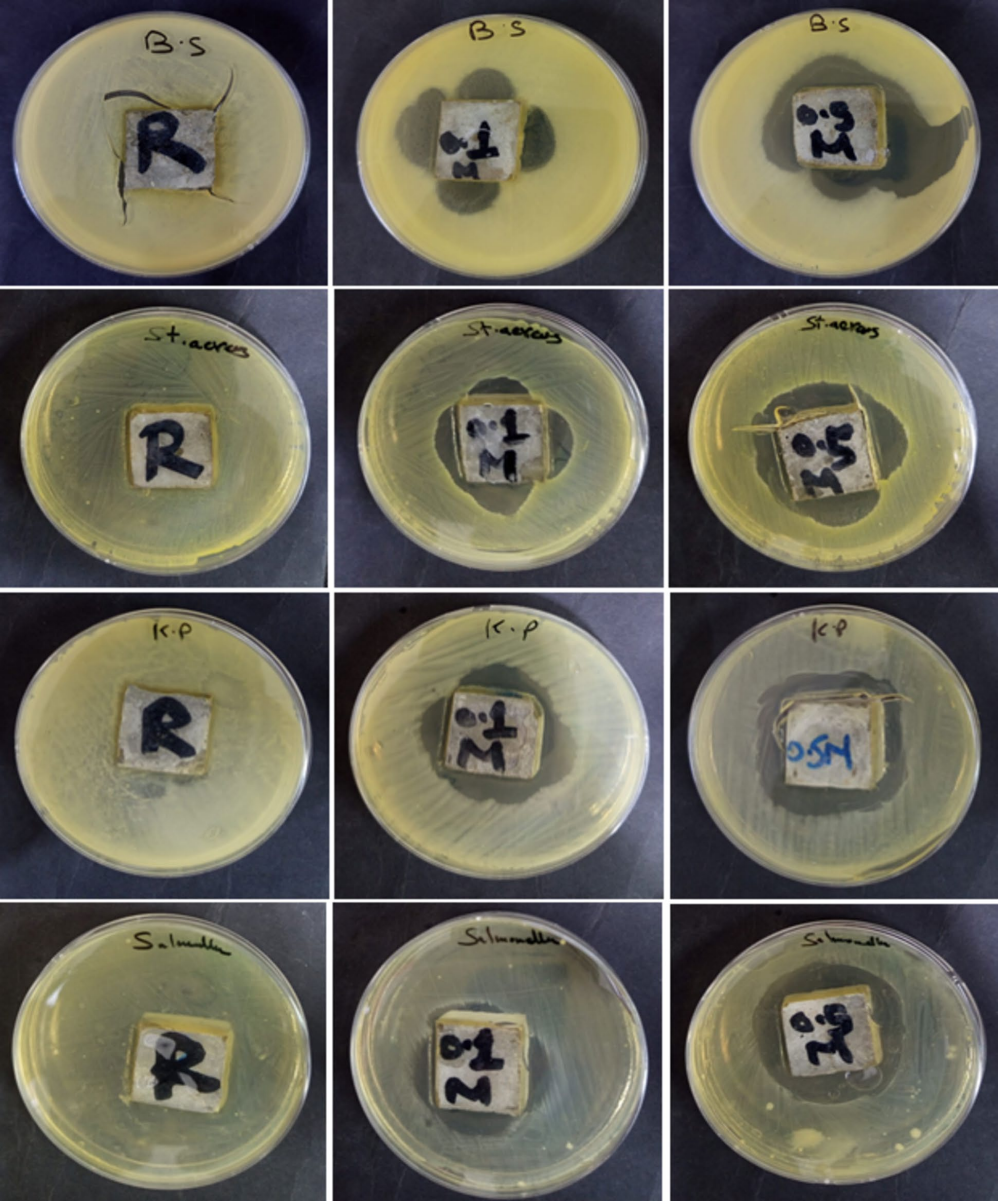
Digital photos for different types of microbial cultures affecting R, R-0.1MOF, and R-0.5MOF discs.

To investigate the multifunctional potential of MIL-53(Al), as innovative additives in ordinary Portland cement (OPC), this study extended the evaluation beyond previously reported enhancements in hydration behavior, microstructure and mechanical strength to include antimicrobial efficacy. The antibacterial performance of MIL-53(Al)-modified OPC pastes was systematically assessed against four clinically significant bacterial strains (*Bacillus subtilis*, *Staphylococcus aureus*, *Klebsiella pneumoniae*, and *Salmonella typhi*) using the agar diffusion method. Cementitious discs prepared with varying MIL-53(Al)dosages (0.1 and 0.5 wt%) were designated as R-0.1MOF and R-0.5MOF, respectively, while free OPC discs served as the control (R). As illustrated in Fig. 11 and summarized in Table 3, the control samples exhibited no inhibition zones, confirming the microbiologically inert nature of neat OPC. In contrast, MIL-53(Al)-modified specimens demonstrated pronounced antibacterial activity, with inhibition zones for R-0.1MOF ranging from 36 ± 3 mm to 42 ± 1 mm across the tested strains. A further increase in MIL-53(Al) content to 0.5 wt% resulted in significantly larger inhibition zones (47 ± 1 mm to 49 ± 1 mm), indicating a clear dose-dependent enhancement in antimicrobial efficacy. These findings underscore the potential of MIL-53(Al) as a strategic additive for developing antibacterial activity and bio-resistant cementitious materials, particularly in environments prone to microbial contamination.

**Table 3:** Inhibition zones (mm) measured in various microbial strains.

Microbial strain	Cementitious disc		
	*R*	*R*-0.1MOF	*R*-0.5MOF
*Bacillus subtilis*-ATCC 6633	NA	40 ± 1	49 ± 1
*Staph. Aureus*-ATCC 6538	NA	36 ± 3	47 ± 2
*K. pneumoniae*-ATCC13883	NA	42 ± 1	48 ± 2
*Salmonella typhi*-ATCC 6539	NA	40 ± 2	47 ± 1

**Fig. 12:**
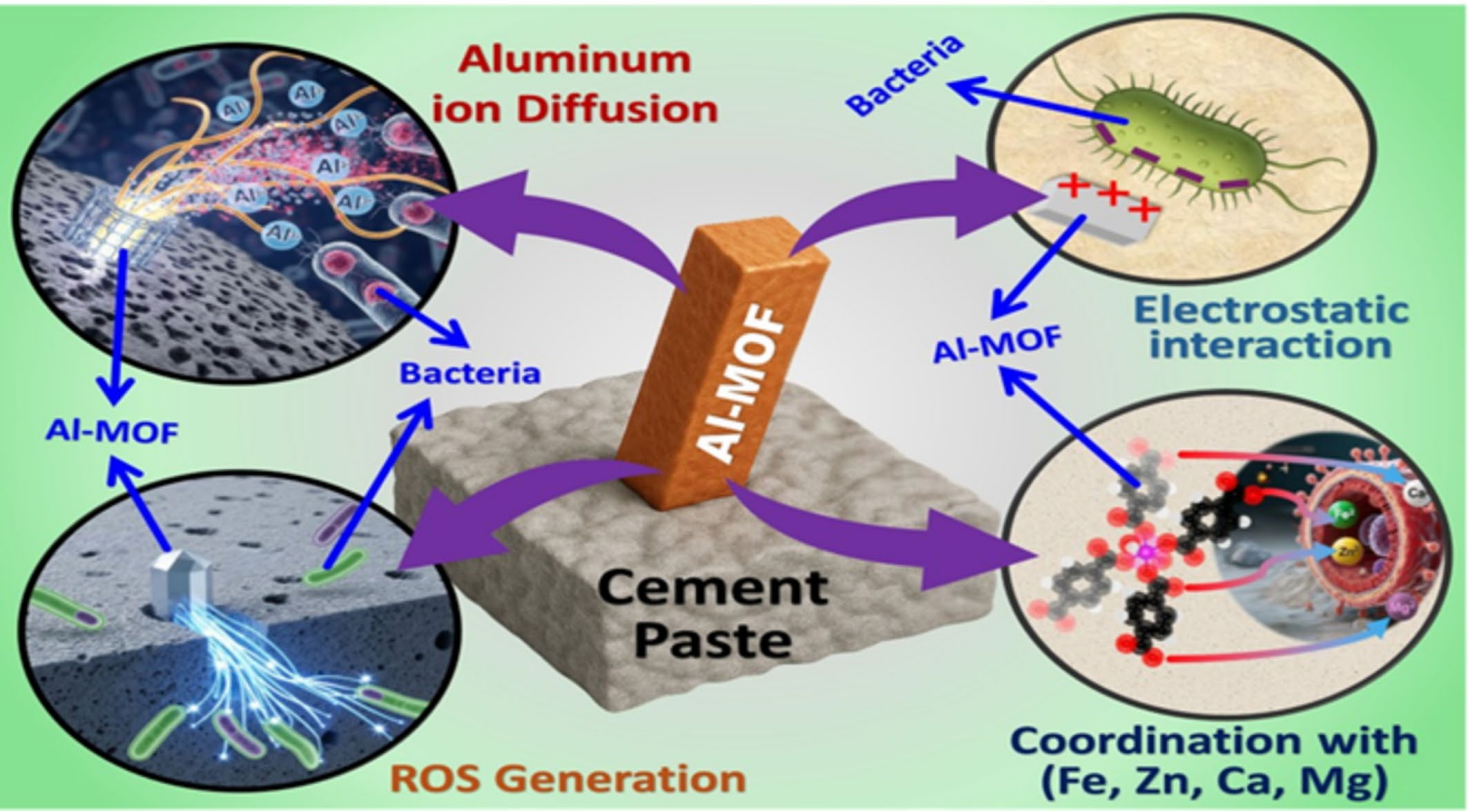
Schematic diagram showing the different mechanisms of R-MOF discs to inhibit bacterial growth.

The integration of MIL-53(Al), an aluminum-based metal-organic framework, into cementitious matrices imparts significant antimicrobial functionality through a combination of synergistic physicochemical mechanisms. Structurally, MIL-53(Al) consists of nanorod-shaped particles formed by aluminum coordination centers linked via terephthalic acid within a tetragonal-like framework, which facilitates its bioactive behavior. Upon incorporation into OPC pastes, MIL-53(Al) exerts antimicrobial effects through four principal pathways: (i) sustained release of Al³⁺ ions, which penetrate bacterial cell envelopes and interfere with vital intracellular processes such as protein folding, enzymatic activity, and nucleic acid integrity^38,82^; (ii) potential generation of reactive oxygen species (ROS) under alkaline cementitious conditions, contributing to oxidative stress and cellular damage^39^; (iii) electrostatic interactions between the positively charged MIL-53(Al) surface and negatively charged bacterial membrane^82,127^ constituents including lipopolysaccharides and teichoic acids resulting in membrane destabilization and cell lysis; and (iv) chelation of Al³⁺ with functional groups on microbial membranes (e.g., thiol, carboxyl, and phosphate moieties), disrupting membrane integrity and metabolic function^42^. Additionally, the terephthalic acid linkers play a dual role by maintaining the structural stability of the framework for sustained ion release and potentially interacting with essential divalent cations (Fe²⁺, Zn²⁺, Mg²⁺, Ca²⁺) that regulate membrane permeability and bacterial metabolism. Nanorod morphology further enhances antimicrobial efficacy by increasing surface area contact and promoting efficient diffusion throughout the cement matrix, thereby enabling prolonged and spatially distributed antimicrobial action^42,44,83^. Figure 12 illustrates the various mechanistic pathways through which R-MOF discs interact with microbial cells, highlighting the multifaceted antimicrobial actions facilitated by the MIL-53(Al) integration within the cement matrix.

### Mechanistic interpretation, comparative studies, and future work

#### Main findings of the present work

The current study demonstrates that incorporating MIL-53(Al) into cement paste notably influences both fresh and hardened properties. The addition of MIL-53(Al) reduces workability and accelerates setting times due to its high surface area and water absorption capacity. Mechanical efficacy is notably enhanced, with the optimum dosage identified at 0.5 wt%, yielding compressive strength improvements of 43.8% and 50.0% at 1 and 28 days, respectively, compared to the control sample. Furthermore, the modified cement exhibits strong bacterial resistance against both Gram-positive and Gram-negative bacteria. Microstructural analyses confirm that MIL-53(Al) acts as a nano-filler and nucleation agent, promoting the formation of additional hydration products such as CSH, C_3_AH_6_, and CASH, leading to a denser and more compact composite.

#### Comparison with other studies

The decline in workability accompanied by the improvement in mechanical performance observed in this study is in good agreement with previously reported findings on MOF-modified cement systems. For example, the incorporation of 0.6 wt% ZIF-67 has been reported to impair flowability while enhancing the 28-day compressive strength by 20.36% ^10^. In a similar vein, the addition of ZIF-8 results in reduced workability alongside a 16.2% increase in compressive strength after 28 days of curing^24^. Likewise, the inclusion of 0.15 wt% Zr(IV)-based MOF-808 produces a measurable improvement in compressive strength, reaching 13.03% at 28 days^23^. Furthermore, MIL-101(Fe) and its amino-functionalized derivative, NH₂-MIL-101(Fe), have been shown to accelerate hydration kinetics, refine the pore structure, and thereby enhance the mechanical integrity of cementitious matrices^26^. Notably, the magnitude of strength enhancement achieved in the present study, reaching up to 50%, surpasses most previously reported values, indicating the superior reinforcing capability of MIL-53(Al). Moreover, in contrast to earlier investigations that predominantly emphasize mechanical improvements, the present work offers a distinctive contribution by integrating antibacterial functionality, thereby underscoring the multifunctional potential of MIL-53(Al) in advanced cement-based materials.

#### Implication and explanation of findings

The obtained results elucidate the multifunctional role of MIL-53(Al) within cementitious systems, which can be ascribed to its ultrafine nanorod morphology, high specific surface area (455 m²/g), and well-developed micro/mesoporous structure. These characteristics enable MIL-53(Al) to effectively occupy capillary, gel, and interlayer pores formed during the evolution of hydration products such as tobermorite (CSH), hydrogarnet (C_3_AH_6_), and strätlingite (C_2_ASH_8_). At an optimal dosage of 0.5 wt%, significant pore refinement is achieved, leading to a more compact and homogeneous microstructure, and consequently enhanced mechanical performance. Furthermore, the hierarchical porous architecture of MIL-53(Al) promotes its function as an efficient nucleation substrate for hydration products, thereby accelerating the dissolution of residual cement phases, including larnite and alite and facilitating improved microstructural development. In parallel, the presence of reactive carboxyl functional groups enables chemical interactions with hydration phases, while the elongated nanorod geometry enhances interfacial load transfer and mitigates crack propagation, collectively contributing to superior mechanical properties. In addition to its structural benefits, enhanced antibacterial activity is attributed to synergistic mechanisms, including the generation of reactive oxygen species (ROS) and disruption of microbial cell membranes. These features position MIL-53(Al)-modified cement as a promising material for advanced antimicrobial and hygienic construction applications.

#### Strengths and limitations

The main strengths of this study can be concluded in the introduction of MIL-53(Al) as a novel MOF additive for cementitious materials, which leads to significant improvement in both early- and long-term mechanical properties, as well as demonstrating strong, broad-spectrum antibacterial activity. On the contrary, the main limitations are that the reduction in workability may limit practical applications without the use of superplasticizers. Performance was evaluated at laboratory scale, requiring validation in real construction conditions. Long-term durability aspects (e.g., shrinkage, carbonation, and chemical resistance) were not fully explored.

#### Conclusion, recommendation, and future direction

In conclusion, MIL-53(Al) is a promising multifunctional additive for cement-based materials, providing simultaneous improvements in mechanical strength and antibacterial properties. The optimum dosage of 0.5 wt% achieves the best balance between performance and material efficiency. It is recommended to combine MIL-53(Al) with suitable admixtures to overcome workability limitations and to explore its performance in concrete systems beyond paste scale. Future studies should focus on long-term durability (shrinkage, chemical resistance, fire, radiation), scaling-up production, cost-effectiveness, and field applications, particularly in healthcare and high-hygiene environments where antimicrobial construction materials are highly desirable.

## Conclusion

This study introduced aluminum-based metal-organic framework nanorods (MIL-53(Al)) as a novel multifunctional additive (0, 0.1, 0.3, 0.5, and 1 wt%) in cementitious systems, demonstrating its capability to simultaneously enhance mechanical performance and impart antimicrobial functionality. The incorporation of MIL-53(Al), synthesized via a modified hydrothermal method, significantly influenced both fresh and hardened properties of cement paste. While workability decreased with increasing dosage, a marked acceleration in both initial and final setting times was observed, indicating enhanced hydration kinetics. Among the compositions investigated, an optimum dosage of 0.5 wt% MIL-53(Al) yielded the most pronounced improvement in mechanical performance, achieving compressive strength gains of up to 50% at 28 days. This improvement is attributed to a combination of mechanisms, including nano-filling, pore refinement, nucleation effects, and improved interfacial bonding. XRD and SEM/EDX confirmed the formation of additional hydration products and a denser matrix, which collectively contributed to enhanced strength. A key scientific contribution of this work lies in demonstrating, for the first time, the dual functional role of MIL-53(Al) as both a reinforcing and antimicrobial agent within cement matrices. The demonstrated antibacterial activity against *Bacillus subtilis*,* Staphylococcus aureus*,* Klebsiella pneumoniae*, and *Salmonella typhi* underscores the potential of this material for advanced applications requiring effective antimicrobial functionality, such as healthcare infrastructure and public facilities. Despite these promising outcomes, certain limitations must be acknowledged. The reduction in workability may necessitate the use of chemical admixtures in practical applications. Additionally, the study was conducted at laboratory scale, and long-term durability aspects, including shrinkage behavior, carbonation resistance, and performance under aggressive environments (acids, sulphates, chlorides) were not fully explored. From an application perspective, MIL-53(Al)-modified cement shows strong potential for use in high-performance and sustainable construction materials, particularly in environments prone to microbial contamination. Future research should focus on optimizing mix design through compatibility with superplasticizers, evaluating long-term durability under real service conditions, and assessing scalability and economic feasibility. Furthermore, extending this approach to mortar and concrete systems, as well as exploring hybrid MOF-based formulations, represents a promising direction for future investigation.

## Data Availability

All data generated or analysed during this study are included in this published article.
